# Illness narratives of people who are homeless

**DOI:** 10.3402/qhw.v11.32924

**Published:** 2016-11-30

**Authors:** Cecilia Håkanson, Joakim Öhlén

**Affiliations:** 1 Department of Health Care Sciences, Palliative Research Centre, Ersta Sköndal University College, Stockholm, Sweden; 2 Department of Neurobiology, Care Science and Society, Karolinska Institutet, Huddinge, Sweden; 3 Institute of Health and Care Sciences, The Sahlgrenska Academy, Gothenburg, Sweden; 4 University of Gothenburg Centre for Person-Centred Care (GPCC), University of Gothenburg, Gothenburg, Sweden

**Keywords:** Illness experience, homeless, interpretive description, narratives, somatic

## Abstract

Multiple illnesses are common in all homeless populations. While most previous studies have focused on experiences of mental illness, there is a scarcity of studies about experiences of bodily illness among people who are homeless. This study aimed to explore illness narratives of people who are homeless, and how homelessness as a social context shapes the experience of multiple and/or advancing somatic conditions. The design was a qualitative single-case study, using interpretive description. Data were generated through interviews, with nine participants who were homeless rough sleepers in Stockholm, Sweden, recruited while receiving care in a support home for homeless people with complex care needs. The findings revealed experiences of illness embedded in narratives about falling ill, being ill, and the future. The particularity of these illness narratives and the way that they are shaped by homelessness give rise to several observations: the necessity of a capable body for survival; chaos and profound solitude in illness and self-care management; ambiguous feelings about receiving care, transitioning from independence, and “freedom” in the streets to dependency and being institutionalized; and finally, the absence of hope and desire for recovery or a better future. The narratives are discussed from the perspective of Frank's four types of illness stories (restitution, chaos, quest, and testimony). The findings stress that to provide appropriate care and support to people who are homeless and have multiple and/or advancing somatic conditions, health care professionals need to be informed both about the individual's biography and about the circumstances under which illness and self-care takes place in the streets.

It is estimated that between 100 million and 1 billion people around the world are homeless (Tipple & Speak, 2005). Multiple—often advancing—illnesses are common in all homeless populations, and chronic conditions are significantly more common than in general populations (Sun, Irestig, Burstrom, Beijer, & Burstrom, 2012). This study focuses on meanings of illness narrated by people who are homeless and have advancing somatic conditions and deals with how these experiences are shaped by a life sleeping rough.

The morbidity panorama of people who are homeless often appears as a complex blend of chronic and acute somatic conditions, for example, chronic obstructive pulmonary disease, chronic heart failure, cancer, neurodegenerative diseases, liver/renal failure, and HIV. Moreover, diseases like diabetes or hypertonia are often poorly treated, resulting in severe health consequences, for example, renal failure, slow-healing leg ulcers, or stroke (Hwang, 2001; Hwang et al., 2001). Other common conditions, especially among people who live in shelters or sleep rough, are tuberculosis, skin diseases relating to parasites (e.g., lice and scabies), and wounds from infections or cuts (Hwang, 2001). Many people who are homeless also struggle with severe mental illness, often combined with the use of illicit drugs and alcohol (Sun et al., 2012).

Accordingly, health-related quality of life has been shown to be rated lower among people who are homeless, especially for those who are rough sleepers, compared with the general population (Fazel, Khosla, Doll, & Geddes, 2008; Sun et al., 2012), and the mortality rate for people who are homeless is four times higher than the average population (Geddes & Fazel, 2011). In line with this, homeless people often have complex care needs, including needs for palliative care, requiring multiple competencies within social service, mental health nursing, and medicine. However, it has been reported that complex care needs due to multiple chronic mental and somatic conditions are significantly associated with increased risk of having unmet health care needs (Argintaru et al., 2013). Studies report barriers to accessing adequate health care, especially for rough sleepers. Among these barriers, the most important seem to be insufficient health insurance and the absence of a regular source of care, for example, not being registered with a general practitioner (Kushel, Vittinghoff, & Haas, 2001; Elwell-Sutton, Fok, Albanese, Mathie, & Holland, 2016). Accordingly, underuse of care prior to death has also been presented (Hwang et al., 2013).

In the context of somatic hospital care, people who are homeless have been described as “difficult” patients that—due to complex care needs, mental illness, the use of illicit drugs, and other circumstances related to being homeless—often have poor treatment compliance and reliability issues in terms of taking their prescribed medicine, maintaining self-care, and showing up to scheduled visits (Hwang et al., 2001; McNeil & Guirguis-Younger, 2012). From the perspective of those who are homeless, however, in studies they have described often being met with lack of respect (i.e., being dismissed or rejected) or understanding about their illness and life situation, on the part of health and social service professionals. Experiences of feeling invisible to health care providers and suffering from being stigmatized as “just another homeless person” rather than being treated as unique individuals have also been disclosed (Drury, 2008; Irestig, Burstrom, Wessel, & Lynoe, 2010; Martins, 2008; McCabe, Macnee, & Anderson, 2001).

Different countries have their own definitions of homelessness (Busch-Geertsema, Culhane, & Fitzpatrick, 2016). In Sweden, which is the context for this study, the definition is broader than in many other countries and built upon four types of situations: ranging from individuals sleeping rough to students being temporarily without housing. However, in this study the focus is on people who had been rough sleepers. Of the approximately 34,000 people (0.4% of the total population) who are homeless or excluded from the regular housing market in Sweden, at least 4000 people belong to this category (The Swedish National Board of Health and Welfare, 2012).

People become homeless for various reasons. The social context of homelessness varies depending on the health and social service infrastructure in a country. In many countries, the welfare system fails to take responsibility when workers fall ill. They often lose their job and eventually cannot pay for housing; hence, homelessness is often the immediate effect of poverty. Another typical scenario is commonly described as a vicious circle of mental illness, illicit drug or alcohol use, broken relationships, and eviction from housing (Watson, 2000; Wendell, 2012). Within Swedish society, homelessness is commonly regarded as an individual social problem rather than poverty, and a substantial proportion of homeless people have additional social problems (e.g., related to drugs and alcohol and mental illness) in addition to the lack of housing per se.

Chronic and/or advancing illness involves recognition of pain and suffering—existential threats that people usually see only as distant possibilities or something that happens to others. Extensive research has proven the experience of chronic and/or advancing illness to be generic in the sense that the taken-for-grantedness of everyday life, and the layers of meaning upon which these rest are profoundly disrupted (cf. Charmaz, 1983, 1995; Delmar, et al., 2006; Pierret, 2003; Thorne et al., 2002). Charmaz (1983) has conceptualized this as a fundamental form of suffering, with loss of self and of body familiarity. An alternative perspective relating to the phenomenological notion of lived body is illness as an existential state of homelessness, an attunement of lived body that has been disrupted (Svenaeus, 2000, 2011). All these theoretical ideas, however, are stated within the context of “ordinary” lives, with people fitting into our postmodern socio-cultural norms and values of what makes a life “balanced or settled.” Most people, at least in western societies, would probably consider a life of homelessness to be disruptive even in the absence of significant illness. With reference to Ricoeur's (1992) philosophy of personhood suggesting that a person is always vulnerable but, at the same time, also capable and resourceful, we anticipate that suffering would outweigh capability in the case of homeless people with multiple and/or severe illnesses.

As has been pointed out previously, Frank (1995) confirms that illness tends to disrupt a person's sense of continuity, identity, and autobiographical coherence. The challenge to the individual, he claims, is to repair the disruption between the body, self, and society. In this way, illness stories attempt to restore an order that has been fragmented by this disruption. Telling stories of illness gives embodied voice to an experience that cannot be expressed in other ways. However, there is a scarcity of qualitative studies providing illness narratives of homeless people with somatic conditions. Most studies have focused on experiences related to mental health and, for example, housing (Tsai, Bond, Salyers, Godfrey, & Davis, 2010), shame/stigma (Corrigan & Miller, 2004), or drugs (Ensign & Bell, 2004), whereas other studies involve experiences of health care encounters (Drury, 2008; Martins, 2008; McCabe et al., 2001; Wise & Phillips, 2013) and palliative care and dying (Song, Bartels, et al., 2007; Song, Ratner, & Bartels, 2005; Song, Ratner, et al., 2007).

Disease always takes place in a life that already has a story, and this story goes on, changed by illness, but also affecting how the illness story is shaped (Frank, 1995). In this study, we aimed to explore illness narratives of people who are homeless and sleep rough, and how homelessness as a social context shapes the experience of multiple and/or advancing somatic conditions.

## Study design and methods

This study is part of a project about care for people who are homeless with complex care needs (Author, 2016). The design is a qualitative single-case study, using the interpretive description approach (Thorne, 2008). This was motivated by our primary focus: to capture experiential variations and commonalities of illness (Thorne, 2008; Thorne, Kirkham, & MacDonald-Emes, 1997) which are of relevance for health and social service professionals who encounter homeless people with severe illness in their clinical practice. By using this approach, we recognize that reality is always complex, contextual, constructed, and intersubjective.

### Study context

The sociocultural context in this case is the homelessness scene in Stockholm, Sweden, and a support home for homeless people from where this project took its starting point (i.e., case sampling) offers qualified care, support, and housing with eight beds. Staff members include social workers, registered nurses, psychiatric aides, assistant nurses, and physicians, all with a range of previous experience working with homeless people or illicit drug or alcohol users; patients with psychiatric disorders; and those with advancing somatic diseases. The purpose for the support home is to provide qualified medical health care and social support to people who are homeless and have complex care needs, to facilitate improved health and social functioning, as well as well-being, and to provide palliative care with two beds allocated for hospice care (Author, 2016; Engel, 2008). The support home allows the use of illicit drugs and alcohol during ongoing care. Another important task is to provide a family-like environment. In this sense, the support home is a “stand-in” home for persons in need of advanced home care, but who do not have a home of their own. For staff, providing a family-like environment entails a certain person-centered approach to care, which we have described in a previous article. In brief, this approach involves striking a balance between being personal enough in the relationship with the patients to facilitate a home-like environment and not becoming too personal, thereby allowing the provision of professional care and support (Author, 2016).

When this research took place (over a period of 6 months in 2014 − 2015), most patients in the support home were men, the mean age of all patients was around 50 years, and all were literate. Most patients had multiple chronic and/or advancing somatic conditions, about one-fifth had psychiatric disorders, and one-third had illicit drugs or alcohol-related diagnosis. Not all patients were rough sleepers.

### Study participants

Staff members (the head of the support home, one assistant nurse, one registered nurse, and one physician) in the support home assisted with the recruitment of participants. As we were particularly interested in the illness experiences of people who were sleeping rough, this was an inclusion criterion. The other criterion was that they should have one or more advancing somatic conditions requiring care. Within these criteria, we aimed to include both men and women with chronic and advancing health problems (i.e., purposeful sampling). For methodological reasons, the participants also needed to be able to communicate in Swedish.

Since the data generation strategy for this study was by interview, the project member who was going to perform the interviews initially spent time in the support home to get to know the environment and to become a familiar face in the support home (see also ethical considerations below) for the patients, thereby facilitating the recruitment of participants. This was based on previous literature about homeless people's experience of distrust and lack of acknowledgement in health care encounters (Irestig et al., 2010; Martins, 2008; McCabe et al., 2001), and on our previous study (Author, 2016), in which staff members emphasized a trustful relationship as being key to successful cares. The staff members suggested and introduced potential study participants. We gave oral and written information about the study, its purpose, and matters of confidentiality, as well as its voluntary nature, including the right to withdraw at any time without needing to give a reason. The persons who agreed to participate gave their written consent prior to the interview.

Seven men and two women, all born in Sweden and between the ages of 45 and 61 agreed to participate. The participants had all been homeless for several years and had mostly been sleeping rough during this time. The information known about family relationships was limited to what they chose to share with us in the interviews: one participant had sporadic contact with his mother and one participant talked about having underage children in foster care. All participants, except one, had been abusers of a variety of hard illicit drugs and/or alcohol (mostly poly drug abuse), and most participants had more than one somatic condition. Most of the participants also had psychiatric conditions. A summary of participants’ somatic health backgrounds is presented in further detail in Table I.

**Table I:** Summary of participant health backgrounds.*

	*n*
Somatic conditions	
Heart failure	3
Diabetes	3
COPD**	1
Cancer	
Head/neck	1
Liver	1
Bowel rupture	1
Infections	
Hepatitis, type B, C and/or D	3
Methicillin-resistant staphylococcus aureus	1
Extended-spectrum beta-lactamase	1
Skeletal injuries	1
Arthritis	1
Walking difficulties: wheelchair bound/crutches	2/1
Impaired hearing	1
Slow-healing leg ulcers/cutting wound	2/1
Colostomy	1
Insulin treatment	3
Mental health-related conditions	
Psychiatric condition	7
Drug and/or alcohol abuse (ongoing)	8
Asperger's syndrome	1
Suicide attempts	1

* Information is based on participants’ own descriptions and on what was available in medical records.

** COPD: chronic obstructive pulmonary disease.

### Data generation

We generated data through individual interviews, entailing a social constructivist position in line with the interpretive description approach (Thorne, 2008), that is., that the data are shaped and, thus, to some extent co-constructed, within the relationship between the researcher and the research participant. Moreover, as Frank (1995) has stated, the illness story is always constructed based on memories that are told, both to another (i.e., the researcher) and to oneself, and the act of telling entails dual affirmation—that the story is worth listening to—as well as reaffirming a sense of “me being there reflecting upon and sharing what I choose to share and remember from my past experiences.” Further, an interview is also spatially shaped and influenced in the setting where it is performed. These interviews took place either in the patient rooms or in one of the social areas of the support home (e.g., TV-room and smoking area) where only the participant and the researcher were present. Before the interview, the interviewing researcher informed the participant again about the study as a whole and about the voluntary and confidentiality aspects in particular. The interviews were digitally recorded and lasted between 25 and 60 min, and a professional transcriber later transcribed them verbatim.

The interviews started with the interviewer asking: “Would you like to tell me about your illness?” followed by, if necessary, more specific questions about how illness affected their lives, how they experienced their body, and how they experienced their role as patients. As fieldwork was progressing, questions were formulated also based on previous interviews with other participants, that is, “When I talked to another participant he told me about situations when … is this something that you have experienced?” Probing questions were also asked such as “I thought about what you said before about … can you tell me some more about that?” “Can you describe a particular situation when … happened?” and so on.

After the first interview, all participants were asked if they would be willing to participate in a second interview. However, even though the majority of the participants initially gave their consent to be approached for a second interview, this turned out to be rather difficult for different reasons, such as being affected by drugs, being exhausted by illness, having unexpectedly left the building despite having a scheduled interview, or being reluctant to participate in a second interview. As a result, only two of the nine persons were interviewed twice.

### Data analysis

An interpretive descriptive analysis seeks to uncover patterns and inherent variations that are characteristic of the phenomenon of interest (Thorne, 2008). In this study, we conducted interviews and analysis using a parallel process, meaning that each interview was discussed focusing on illness-related topics that were brought up by previously interviewed participants (e.g., experiences of self-care). Such topics then inspired the following interviews, further developing the topic in question (cf. Thorne, 2008).

The procedure for analysis started with listening to the narratives and reading the transcripts, to get an overall impression of the nature of the data. The narratives were then broadly coded and organized into clusters of linked themes, for example, narratives about falling ill and seeking help. During this process, we noted analytical questions, comments, and possible interpretations. These clusters of broadly coded text were then further analysed and interpreted in the context of the overall understanding of the descriptions (i.e., in what situations, activities, and so forth, the descriptions were embedded) by continuously moving back and forth between the clusters and the texts as a whole. Of particular interest was whether and how the narratives were characterized by homelessness, that is, whether the participants considered the situations or activities to be an ingrained part of their homeless life. This process also involved moving back and forth between the data, the analytical notes and theoretical and empirical literature. Relevant previous empirical work supported the interpretations, whereas theoretical reasoning—for example, about the nature of illness narratives (Frank, 1995)—supported further understanding and interpretation of the data (Thorne, 2008). In the final interpretative step, we merged the analysed clusters of text according to their interpreted meaning, thus representing the variations that together shaped the patterns we present as findings.

We assured credibility by applying the principles of an interpretive description study, namely, epistemological integrity—reviewing previous literature or knowledge and taking this into account when designing the study; representative credibility—striving to include both men and women of various ages to gather variations in illness experiences, albeit without claiming to cover all the possible variations; and finally, analytic logic and interpretive authority—by continuous exchange of reflective and critical reasoning within the research team about interpretations. Moreover, alternative interpretations were discussed, with the purpose of confirming or rejecting the original interpretation before closure of the analysis process (Thorne, 2008).

## Ethical considerations

Conversations with ill persons always need to be performed with great flexibility and respect towards the consent of the individual so that the researcher's presence is not experienced as intrusive or a threat to the person's integrity (Addington-Hall, 2002). The voluntary nature of participation and the right to withdraw from the study at any time were emphasized (Beauchamp & Childress, 2008; World Medical Association, 1994). Before starting the recruitment, the interviewer spent time in the support home to become familiar with the context and to become a familiar face for both the patients and staff. The head of the support home advised this on account of the fact that (due to previous bad experiences with health care, social care, and other authorities) many of the homeless persons were very distrustful, and building trustful relationships had proven to be the key to successful care in the support home.

During the study, staff members were notified about when participants were interviewed. The interviewer made a special effort to be attentive to participants’ emotional reactions during the interviews, and the participants were encouraged to let the interviewer know if they felt uncomfortable. They were also encouraged to talk to any of the staff if the interviews generated feelings/emotions that they felt a need to talk about at any time after the interviews. The Regional Ethical Review Board in Stockholm, Sweden, approved the study prior to commencement (no. 2013/1548-31/4).

## Findings

The illness narratives in this study, and the ways that they were shaped by homelessness reveal patterns of varying experiences that refer to different time points during the illness trajectory. For the sole purpose of structuring the findings, these temporal patterns have been given the following descriptive subheadings: *falling ill*, *being ill*, and *the future*. It is important to note that the participants’ narratives were fragmented and disordered and not explicitly told within a temporal order. Hence, these time points reflect a structure to organize the participants’ stories that facilitate interpretation of meanings and not a specific process of illness in homelessness.

### Falling ill

Falling ill refers to the point in time of the participants’ illness trajectory when their condition was poor and deteriorating, eventually leading to health care seeking, and includes how everyday life on the streets sleeping rough influenced this. When talking about falling ill, they highlighted two major aspects. The first—having a “capable body and a strong mind”—was presented as critical for survival, and the second—having no one else but yourself to rely on—was part and parcel of being homeless. These two aspects were related and hence, overlapping in the narratives.

All participants had multiple morbidities and a body that they described as “bruised,” as a result of their difficult lives, involving drug abuse; sleeping rough under non-hygienic, uncomfortable conditions; not having access to proper clothes, shoes, or nutritious food; fighting; and so on. Yet a capable body was described as being their most important “tool” for getting through the day. Falling ill meant being vulnerable in the streets, and therefore, listening to and acting upon bodily signals was crucial but often difficult. One woman, who had a severe intestinal disease that had recently required surgery and a colostomy, said:

That's something that I never once for a second let go of you know, to ignore my own signals, from my own body. When my body has signaled that something is wrong, I have listened to it, and most of the times, well, sometimes anyway, I've been right, more or less. At least that something wasn't normal.

At the same time, this woman said that being addicted to illicit drugs meant the risk of losing control and losing body awareness and thereby the ability to look after herself and her bodily illness needs (e.g., to appreciate symptoms of illness and seek help accordingly), and she had to recognize this risk and fight against it:

When the body breaks, you need to reflect mentally upon your life situation and your self-responsibility to master the situation: you're in a constant battle between your bodily signals and your mental desires [for drugs]. It is really difficult to balance, like getting the mind and the body to speak the same language. It is a weighing scale, like this [demonstrates by wobbling her hands], it takes a lot of experience. And you know, like to learn about your own body, especially when you live the kind of street life that I do is really [paus] … I have a lifetime of drug abuse experience, so I know how important this is.

Other participants also described having developed a certain mental strength over the years: “If you have that mental strength, you will survive most situations out there. Like, it is an ability to survive kind of, and to stay calm on the inside—the minute you let go of that, it all goes south.” One man, who had been living on the streets for most of his adult life, who had encountered many ill fellow homeless persons, described homeless people as “persons without parachutes,” meaning that giving in to illness means dying because of the lack of a safety net of friends and family to turn to in times of need, or, for example, a welcoming bed in a hospital, or a place to take care of your needs:

It is as hard as hell. Like, here [the support home] you have all your stuff in your room and you have a bathroom and a shower and all. When you're out there [in the streets] you have to carry it [bandages, soap, clothes.] around all the time. That's pretty hard. And not getting the warmth [temperature] either. That's really tough.

In such circumstances, the homeless person must overcome the situation no matter how ill they may feel. Another man, who had severe problems with his legs said: “You can't just lie down because of this and you know, like, now I can't walk… cause I mean, no one else is going to walk for me, But the fact is, If you can't walk you're gonna die out there.”

The participants’ relationships with significant others had long since been compromised. Whereas some talked about friendship with people they come to know on the streets, this was not friendship in the sense of friends who are there in urgent times of need, and the predominant experience was that of being alone and being able to rely on no one else but yourself. Life had forced them to become independent. One man, who had spent several years in a camping spot, said:

You know, I am a cabin dweller and we don't ask each other for help with these things [referring to wound cleaning as an example]. It's just if you're more or less half dead and can't get out of bed, then you have to knock on the floor or send morse-signals. Like, you have to stay independent.

This man also talked about how the changed demographic situation of homeless people in the city, with increased numbers of immigrants moving from one place to another, had negatively affected the sense of connectedness and community among homeless persons: “Life as homeless used to be a life where you encountered death much more often, but now with so many places where people stay there is a constant movement, so you don't look after each other much anymore.” Thus, this changed demography had made life in the streets more anonymous, especially in times of advancing illness and dying.

The participants’ previous bad experiences of encounters with health care providers, combined with neglect of bodily signals, usually resulted in delays in seeking help: “I was in the street when I was worst off. Until my legs wouldn't carry me anymore. Then someone called an ambulance and that was at the last minute really.” One woman, who was urgently transferred to hospital because of an intestinal rupture said:

P: No, and I mean like [paus] … I had this thing for over a month and a half, and I had no idea what the fuck this was all about. Imagine yourself running three marathons in one night, that's how much soreness I had in my legs after walking a few meters. I kept falling all the time, I was in such bad shape.

I: Where were you then?

P: I was in this shelter and I had just about lied down and it all came out the wrong way [referring to faecal content coming out of the mouth]

I: I see.

P: Yes, you know there were blue lights and sirens. When I came to the hospital, I had no blood pressure or pulse they said, and a temperature of 43 degrees.

Like this woman, most participants equally described their ending up in hospital to have been urgent or last minute and under somewhat chaotic circumstances.

### Being ill

Being ill refers to a point in time when the participants experienced moving from being independent and living “free” in the streets, to becoming dependent and institutionalized. It illustrated how previous encounters with and distrust from society in general, and the health and social service in particular, shaped their journey from relying on no one to being forced to depend on others for their well-being. Moreover, it involved being temporarily housed and cared for, in a somewhat unusual institutional environment like the support home, with rules and regulations, but also with professional love and attention.

Being bodily ill with a disease needing self-care normally requires facilities that those sleeping rough do not have access to—a clean environment, fresh water, soap, first aid, and privacy. The woman with the colostomy talked about the misery she found herself in when trying to manage her condition, while at the same time being on drugs. She felt undignified and dismissed by lay people who she asked for help, in the streets, in restaurants, and so on. She said:

P: It has happened to me several times, like I've been somewhere in the city, and the ostomy pouch has ruptured, and no one has offered me a place to go to a toilet so I can fix it. When you're standing there in the middle of the night in a short dress and the pouch—you can feel the shit starting to pour down on your stomach and on your legs. I've been so bloody angry sometimes that I've just changed the bag where I stand, in the street. I've undressed, just to protest, to show, that you don't treat people like that.

Because it's pretty fucking hard walking in the streets and that bloody [ostomy] bag like, explodes. It's… you see these judging faces, and sometimes it's more than I can take.

I: Who are these judging faces?

P: Just people.

In their encounters with health care services, all participants expressed having been neglected and rejected because of their homelessness. One man recounted having been troubled by a urinary infection for over a year. His experience was that, because of his biography, he was neglected and not given adequate care:

P: And I have been walking around with this [urinary problems] for a year.

I: That's a long time …

P: Yes, well, I thought it was my medicines [for other conditions] that made it worse, and I mean, it smelled like shit, the pee. And, the second day I came [to the emergency department] it was like, yeah, you have a urinary infection, and then I got antibiotics. Do you get it, a simple thing like that they just ignored in the xxx [name of outpatient clinic for drug abusers], just because you're an old addict and drunk, yeah, that's the way it is.

Most participants had similar stories to tell, about being stigmatized and rejected “by default” because of their previous history of drug or alcohol abuse; “When they [the health care professionals] turn on the computer you automatically become a drug addict, not a human being.” Another woman said that seeking help felt like “being forced to seek asylum in your own country,” and she continued:

You need a staff member to advocate for you against staff in other health service settings, who patronize, belittle or use their power to remind you of who/what you are and, e.g., refuse to give you pain killers or other medicine.

The reason for refusing to give the woman painkillers, she explained, was that because she was a drug abuser, they automatically assumed that she was trying to take advantage of the situation, that is, to obtain free drugs. Another man said, “An alcoholic can have loads of pain killers to feel all right, but a drug abuser has to lie in his own shit and vomit and can barely get paracetamol.”

Sometimes the participants’ anticipation of being stigmatized also made them misinterpret situations. For example, one participant talked about being isolated in the patient bedroom in the support home, to prevent contamination from contagious bacteria, and how this created a sense of violation of his freedom, and a feeling of being banished or excluded:

That feeling, it is still there you know. Because of my intestinal bacteria, I have to eat in my room. I am sort of banished to my room. They come and serve me. And according to the law, it says that as long as you stick to the hygiene and clean your hands and the toilet, you're all right. Then you can, like, I can go to McDonalds and there no one complains, but as soon as I get in here, it's just … it's wrong.

Hence for these individuals, routine restrictions like those described did not only impose on their sense of freedom, but also—given their vulnerable position and previous experiences of stigmatization and judgmental attitudes from health care professionals—could be misinterpreted as “punishments” or “special treatment” on account of them being homeless.

The participants described that being institutionalized—in this case, in the support home—enabled comfort and dignity, and a sense of being treated as any other people, getting all the help they needed without having to beg for it. One man illustrated this:

It has required a bit of adaption here. You know I have been locked up in prison for thirty years. I have been forced to learn to accept help. I have never done that before, ever. And it works here actually. The staff, they are quite perceptive so they can see when my pain is bloody awful. And I can accept that they help me, they don't pity me. They treat me as a normal person and that is not so common for me.

The participants clearly recognized the difference between the caring environments in the support home, which was described by most participants as a “warm atmosphere like you've never experienced before,” and their previous experiences of being misrepresented and judged in hospitals: “They just treat you so bad [refers to other health care experiences]. It's like they have these preconceptions and prejudices about you.” However, at the same time, they talked about the support home feeling like an alien place with rules and where freedom was lost because of their new dependency on others. Another man talked about feeling troubled by the burden of his broken body in the sense of becoming dependent on others and being offered help in the support home:

I feel as if my body is broken. Every time I walk from here [the bed] to get food in the kitchen or if I need to carry something, I can't do it with the damn crutches. It's impossible to do anything, and it is really annoying when people want to help you all the time.

The participants could also clearly sense that there were boundaries between them and the staff in the support home, which challenged and hindered the participants from getting the “homey” feeling, which was part of the goal of care in the support home:

It's like, it's OK to come in to the kitchen but not OK to start doing stuff on your own without asking, like making a cup of coffee for example. And it's OK to sit in the TV-room and talk about stuff that happens on the TV, sort of, but it's not OK to ask about their [referring to health care professionals] life.

Hence, prohibition from entering the kitchen to take something out of the refrigerator or crossing the boundaries of the privacy of staff by sitting too close, asking private questions, were experienced as clear markers, separating them from others in the sense of not being equally valued.

### The future

The final time point of the illness trajectory represented was the future. These narratives comprised differences in the participants’ ways of viewing their illness and life situation as a whole, and how these circumstances influenced both their hopes for the future and their thoughts about death.

The interviews opened up a space for the participants to voice their illness experiences and to share aspects of their biography. Although all participants generously shared their experiences, they at the same time disclosed ambiguity in doing so, and they talked about not seeing the point in talking about either their previous or current life situations. They said that the only thing that talking about their life would do was to bring back painful memories of a broken life and remind them of all their failures: “It's too hard to think of all the shit in my life, it will do nothing good for me at all” and, hence, talking about the past would do nothing to help the future—as they could not imagine that their lives would ever be any different:

It doesn't end just because you end up in health care. It never ends you see, nothing ever ends. And the disease only gets worse. The cancer in my liver will remain, and all the cysts in my arms will remain.

Just like this man, all participants explained that the future was not something to look forward to or to have hopes for. They knew a lot about being ill on the streets from their own experiences and from other fellow homeless persons who they had met over the years. As a result, they reported that, with no hope of recovery from illness or of a changed life situation, drugs and alcohol would continue to provide them with comfort, despite knowing that they would worsen their illness. One man said:

You might as well kill us, it doesn't really make a difference. No… my situation is, … I just have to continue. It's hard to buy alcohol on Sundays [liquor stores are closed in Sweden on Sundays]. No, I will not quit that. I have no reason to. I don't have anything to look forward to anyway.

When the participants were asked how they felt or thought about death and dying, they explained—in a rather rational manner—that their current life as homeless people was without meaning and therefore death was nothing to fear. They reported quite the opposite that death would mean being freed from humiliation and from being let down by people and society all of the time: “Well, if you ask about the meaning of death, it is to be freed from humiliation. That can be a kind of life too, to be freed from all that shit.”

## Discussion

In this study, we have interpreted illness narratives among people who are homeless rough sleepers, with multiple and/or advancing somatic conditions. The particularity of these illness narratives and the way that they are shaped by homelessness within their specific Swedish context give rise to several observations: the necessity of a capable body for survival; solitude in illness and self-care management; ambiguous feelings about receiving care, transitioning from independence and “freedom” in the streets to dependency and being institutionalized; and finally, the absence of hope and desire for recovery or a better future

The narratives of our study participants were fragmented and disordered. Frank (1995) argued that people who are seriously ill have lost their sense of temporality; the present is not what the past was supposed to lead up to, and the future is barely thinkable, and hence, the illness story becomes jumbled. Moreover, the telling of the story relies on the retelling of what is remembered, that is, the illness narratives are constructed, based on what memories can be recalled.

Frank (1995) put forward four types of illness stories: restitution, chaos, quest, and testimony. He claims that all types of stories are present within narratives of people who are ill, but at different times and in different situations, and either of the types may be more or less in the foreground. The narratives in this study were clearly distinguished as chaos stories that were built up around the participants’ day-to-day life, which at least to us seemed to be immersed in chaos, related to finding places to sleep, getting drugs, finding meals, and so on, and of course, dealing with illness. The chaos narrative is according to Frank (1995) the most embodied story, meaning that events are told as embodied life experiences: without sequence or discernible causality.

Frank (1995) claimed that restitution narratives dominate most people's illness narratives, since most people want to be healthy again. He also affirms that the ill person's own desire for restitution is compounded by the contemporary expectation that other people want to hear restitution narratives. They are the culturally preferred narratives. The confrontation with mortality cannot be part of a restitution story, because this implies having the self-story end before the life is over (Frank, 1995). However, people with advancing illness do also question restitution and seek other types of narrative structures. Several examples of this are to be found, for example, within the context of cancer (cf. e.g., Leveälahti, Tishelman, & Öhlén, 2007; Öhlén & Holm, 2006).

The narratives of this study gave very little impression of restitution. A reasonable interpretation could be that in homelessness contexts such as the one we have described, a gradual breakdown of the body is inescapable, and that expecting or hoping for a better future is beyond imagination. While restitution stories presuppose that control is necessary to effect restitution, chaos stories presuppose lack of control, and hence, the chaos narrative is beyond a “way out” (Frank, 1995). We would assert that this is supported by the ways in which the participants talked about destructive behaviors (e.g., continuing with alcohol and drugs even though they knew that this would worsen their condition) and the body as being worn out and beyond recovery and self-management, as well as them feeling that there is no place for a diseased body in the streets.

The participants in our study had chronic, multiple and/or advancing somatic conditions, and hence, they belonged to a group eligible for care according to a palliative approach, that is, relief of symptoms and the enabling of well-being and quality of life despite illness (cf. Sawatzky et al., 2016). Within the palliative context, one of the key goals is to enable people with advanced illness who are dying to “live until they die.” This entails several dimensions such as helping people to find meaning in life until the end (Randall & Downie, 2006; Sawatzky et al., 2016). It goes without saying that, in the context of homelessness, this is both challenging and worth reflecting upon. The participants in this study described the care received in their support home as being comforting and reported that it had enabled feelings of being treated with dignity as “insiders” of the established society. This may be one way of enabling people with such complex illness and disrupted biographies to find meaning, and these narratives may actually reflect this, even though the participants do not refer to meaning-making per se. An alternative interpretation is, of course, that this is their way of self-presentation, that is, the performance alternative —to disclose their wishes or desires for a future life that they have not managed to uphold before in their lives—would be (potentially) embarrassing and unthinkable. This corresponds well with the phenomenon of self-devaluation that has been previously described within narratives of identity of homeless people (Boydell, Goering, & Morell-Bellai, 2000).

Hydén (2008) has brought to the forefront the idea that, among certain groups of individuals with communication and/or cognitive deficiencies (e.g., dementia or mental illness), the narratives are often broken in the sense that telling a coherent story may be difficult under such circumstances. This links to the narrative as an expression and construction of identity, that is, being “me” despite illness. In chaos stories, the body is so degraded by an over-determination of disease and social mistreatment (which was characteristic of the narratives in this study) that survival depends on self-dissociation from the body (Frank, 1995). The participants never talked about the body as lived; in the narratives, there was always a dissociation from self. That is, they talked about their bodies and their body parts as broken, tired, worn out, hurting, and incapable. This phenomenon is not exclusive to this context of homelessness. The literature voicing illness and discussing this from the lived body perspective is extensive (cf. the work of Finlay, 2006; Toombs, 1988). However, the context from which these experiences unfold and the implications of these broken bodies, related to this context, are clearly novel.

Ashworth (2003) has offered a heuristic understanding based on life-world fractions, interlinked analytical aspects of a larger whole that embody the individual. Two of these fractions are selfhood and sociality. Selfhood refers to what a situation means for social identity; the person's sense of agency, and their feeling of their own presence and voice in the situation. Sociality is the perceiving of the body of another person as a prolongation of one's own intentions, a familiar way of dealing with the world, given that the culture is shared. Relating these ideas to our narratives, the first thing that comes to mind is the self-presentations of the participants, and the fact that they were in such an unfamiliar context of being both housed in the support home and being interviewed. It was a highly unusual situation for them to be encouraged to voice their narratives and to have the precedency of speech in these conversations. Moreover, as patients, being in this environment meant being in an unfamiliar social culture, together with people upon whom they now depended but could not identify with socially. Finally, their previous experiences of similar contexts were of stigmatization, inferiority, and rejection. Hence, the extent to which these narratives were “authentic” (if such narratives exist) is impossible to say. One can argue that this is merely a methodological issue. However, as in any qualitative study, it is always the choice of context that creates the conditions and shapes the nature of data. It may be that, for the participants, being hospitalized in the support home created a distance from their everyday world, which opened a narrative space for them to voice and reflect on their lives and illnesses. The second aspect related to selfhood that comes to mind links to Ricoeur (1992) and his notion of the person as being both suffering and being capable at the same time. Having a sense of agency means feeling that you have the power to influence your situation. This is linked to the participants’ capability to rise until they finally have to give in to their illness. Capability may in fact be their greatest survival tool. Being ill, on the other hand, can be interpreted as a state in which they have lost their sense of agency.

### Methodological reflections

Telling narratives in interviews, as in any social context, involves considerations regarding preformativity. The narratives in this study are based on how the participants choose to present themselves and what they choose to share. This is in line with any qualitative study, but considering the conditions under which these participants have existed for most of their lifetime since being young, including a drug-taking environment, together with their history of distrust of others, this may have influenced their narratives.

Moreover, these narratives were told in conditions, which were not the norm for the participants, being severely ill and being housed and in a safe, structured environment. The influence of drugs or alcohol is not unusual for the participants, but having the kind of attention that an interview situation offers is. Hence, the narratives are told outside of their social context, and so one could argue that they are constructed based on memories of their familiar surroundings, which is now reflected upon in relation to this new unfamiliar context, and this may, or may not, have shaped the content. However, it should be noted that, to our knowledge, there is no other existing study about this particular topic. Moreover, we do not make any claims to have covered “all” possible variations of experiences/narratives (Thorne, 2008), and we are aware of the many different social contexts of homelessness around the world. Hence, we believe that this study provides novel perspectives and understanding, which are of relevance for all health and social service professionals who encounter people who are homeless and ill.

## Concluding reflections

In the illness narratives of rough sleeping homeless people that were interpreted in this study, some particularities were found that we argue are shaped by homelessness. The notion of what a functioning body is seems to be relative to circumstances and so does vulnerability, capability, and suffering. In fact, all these aspects that we consider when dealing with illness in a general sense seem to be relative from the point of view of a homeless life. The chaos and solitude in illness and self-care management are two examples of what characterizes illness within the context of being a homeless person sleeping rough. The uncertainty involved in becoming hospitalized and receiving care is another. Hence, it is not the nature of the illness experience itself that is different, but the social circumstances in which the experiences take place. The lesson learnt is that, to provide appropriate care and support to people who are homeless, health care professionals need to be informed both about the individual's biography and about the conditions under which illness and self-care takes place on the streets. This can be facilitated by, as in any health care situation claiming person-centeredness, inviting the individual to share his/her biography and current life circumstances. Interventions involving person-centered flexible models for providing care and support, including ambulatory solutions and adequate self-care support, that appreciates the homeless person as a resourceful agent, are suggested—for practice and for research evaluation.
